# The *Chlamydia trachomatis* secreted effector TmeA hijacks the N-WASP-ARP2/3 actin remodeling axis to facilitate cellular invasion

**DOI:** 10.1371/journal.ppat.1008878

**Published:** 2020-09-18

**Authors:** Robert Faris, Alix McCullough, Shelby E. Andersen, Thomas O. Moninger, Mary M. Weber

**Affiliations:** 1 Department of Microbiology and Immunology, University of Iowa Carver College of Medicine, Iowa City, Iowa, United States of America; 2 Central Microscopy Research Facility, University of Iowa Carver College of Medicine, Iowa City, Iowa, United States of America; Purdue University, UNITED STATES

## Abstract

As an obligate intracellular pathogen, host cell invasion is paramount to *Chlamydia trachomatis* proliferation. While the mechanistic underpinnings of this essential process remain ill-defined, it is predicted to involve delivery of prepackaged effector proteins into the host cell that trigger plasma membrane remodeling and cytoskeletal reorganization. The secreted effector proteins TmeA and TarP, have risen to prominence as putative key regulators of cellular invasion and bacterial pathogenesis. Although several studies have begun to unravel molecular details underlying the putative function of TarP, the physiological function of TmeA during host cell invasion is unknown. Here, we show that TmeA employs molecular mimicry to bind to the GTPase binding domain of N-WASP, which results in recruitment of the actin branching ARP2/3 complex to the site of chlamydial entry. Electron microscopy revealed that TmeA mutants are deficient in filopodia capture, suggesting that TmeA/N-WASP interactions ultimately modulate host cell plasma membrane remodeling events necessary for chlamydial entry. Importantly, while both TmeA and TarP are necessary for effective host cell invasion, we show that these effectors target distinct pathways that ultimately converge on activation of the ARP2/3 complex. In line with this observation, we show that a double mutant suffers from a severe entry defect nearly identical to that observed when ARP3 is chemically inhibited or knocked down. Collectively, our study highlights both TmeA and TarP as essential regulators of chlamydial invasion that modulate the ARP2/3 complex through distinct signaling platforms, resulting in plasma membrane remodeling events that are essential for pathogen uptake.

## Author summary

*Chlamydia trachomatis* is the etiological agent of trachoma and is the most common bacterial sexually transmitted infection worldwide. As an obligate intracellular pathogen, bacterial proliferation only occurs within the confines of a host cell, thus invasion is the critical step in the infection process. While the secreted effector protein TmeA is known to be essential for invasion and bacterial pathogenesis, how TmeA promotes invasion was previously unknown. Here we show that TmeA binds to the GTPase binding domain of the nucleation promoting factor N-WASP and their interaction is necessary for recruitment of the ARP2/3 complex to the bacterial entry foci. Moreover, our study shows that while the secreted effector protein TarP is also involved in recruiting the ARP2/3 complex, it functions in a pathway that is distinct from N-WASP. Collectively, our study suggests that TmeA and TarP target distinct cell signaling pathways that converge on the ARP2/3 complex, a key regulator of pathogen uptake.

## Introduction

*Chlamydia trachomatis* is an obligate intracellular pathogen that is the leading cause of infectious blindness and is the most common bacterial sexually transmitted infection worldwide [1]. Despite the significant impact chlamydia has on global human health, we still lack a fundamental understanding of the most crucial step in the establishment of infection, invasion of the host cell.

All chlamydiae exhibit a biphasic developmental cycle in which the bacteria alternates between two forms: an infectious elementary body (EB) and the replicative reticulate body (RB) [1]. Following invasion of non-phagocytic cells, the EB resides in a membrane bound endosome-like compartment, termed the inclusion. The inclusion quickly diverges from the default endocytic pathway and instead traffics along microtubules to the peri-Golgi region where the EB differentiates into an RB and replication ensues [2,3]. Following completion of the developmental cycle, the RBs undergo asynchronous conversion into EBs which are then released by host cell lysis or extrusion, allowing the infection cycle to begin anew [4].

As an obligate intracellular bacterium, *C*. *trachomatis* cannot complete its replicative cycle independently and is completely dependent on a host cell to provide a replicative niche conducive to bacterial replication. Similar to other Gram-negative bacteria, chlamydia possesses a type III secretion system (T3SS) that it uses to translocate bacterial virulence factors, termed effectors into the host cell [5]. At the end of the developmental cycle, *C*. *trachomatis* pre-packages select effector proteins into the T3SS apparatus, priming the EB to initiate new rounds of infection [6,7]. Evidence suggests that some of these effector proteins must be translocated into the host cell and successfully interact with host proteins to modulate endocytic dynamics and initiate uptake of the bacterium. Translocated membrane effectors A and B (TmeA and TmeB), translocated actin recruiting protein (TarP), and translocated early phosphoprotein (TepP) are all delivered into the host cell prior to pathogen entry and are thus presumed to be crucial for successful host cell infiltration [6]. Although the literature has reported on some aspects of each of these effector proteins, including putative binding partners, their precise roles in chlamydial uptake remains ill-defined. TarP binds to Rac guanine exchange factors (GEFs) Sos1 and Vav2 which activate Rac, presumably to activate the ARP2/3 complex via the nucleation promoting factor (NPF) WAVE2 [8,9], although this has yet to be demonstrated during invasion. TarP can also potentiate cytoskeletal rearrangements through direct nucleation and polymerization of actin [10,11], although this has yet to be directly linked to plasma membrane remodeling during chlamydial invasion. Recent advances in chlamydial genetics confirmed that TarP is indeed important for *C*. *trachomatis* invasion of nonphagocytic cells [12,13]. TepP binds and recruits the scaffolding proteins CrkI-II and phosphatidylinositol 3-kinase to entry foci, however TepP null mutants are not impaired in invasion [6,14]. TmeA has been shown to be necessary for bacterial invasion and pathogenesis [15,16]; however the mechanisms utilized by TmeA to promote invasion are unknown. TmeA was previously shown to bind a large scaffolding protein, AHNAK, yet depletion of AHNAK does not impair bacterial invasion and AHNAK is still recruited to the entry site in the absence of TmeA [15,17]. Thus, TmeA’s role in invasion is independent of its interaction with AHNAK and the physiological relevant host targets of TmeA were unknown prior to this report.

Nucleation promoting factors (NPFs) such as N-WASP stimulate the ARP2/3 complex, promoting actin nucleation and branching required for filopodia formation, membrane ruffling, membrane invagination, and endocytosis [18]. Intramolecular interactions between the GTPase binding domain (GBD) and C-terminal WH2-connector-acidic (WCA) domain of N-WASP autoinhibit its NPF activity [18]. Binding of the small GTPase Cdc42 to the GBD of N-WASP induces a conformational change, freeing the WCA region to interact with the ARP2/3 complex [18,19]. Using molecular mimicry, the *Escherichia coli* T3SS effector protein EspFu circumvents the need for Cdc42 by directly perturbing N-WASP autoinhibition to promote actin assembly, which facilitates translocation of additional T3SS effectors into the host cell [20–22]. The *Shigella flexneri* virulence factor IcsA similarly binds to the GBD to relieve N-WASP autoinhibition, allowing recruitment of the actin machinery necessary for actin-based motility and cell to cell spread [23,24]. While pathogenic manipulation of N-WASP has been suggested to be necessary for bacterial invasion by *Salmonella enterica* serovar Typhimurium [25] and *C*. *trachomatis* [26], how intracellular pathogens hijack N-WASP function to promote invasion is ill-defined.

Here we show that the *C*. *trachomatis* effector protein TmeA binds N-WASP and colocalizes with the ARP2/3 complex correlating with plasma membrane perturbations at the site of chlamydial entry. We demonstrate the necessity of the ARP2/3 complex and N-WASP for chlamydial invasion and show that TmeA interacts with N-WASP through binding of the GBD region. Significantly, our results indicate that TmeA targets the ARP2/3 complex through a pathway that is distinct from TarP. Thus, we propose that TmeA activates N-WASP, which subsequently activates the ARP2/3 complex to stimulate actin branching events essential for remodeling the plasma membrane to facilitate chlamydial uptake. Our findings shed new light on how this important human pathogen induces its own entry into host cells.

## Results

### TmeA possesses a C-helix GTPase binding domain ligand motif that binds to the GTPase binding domain of N-WASP

**Fig 1:**
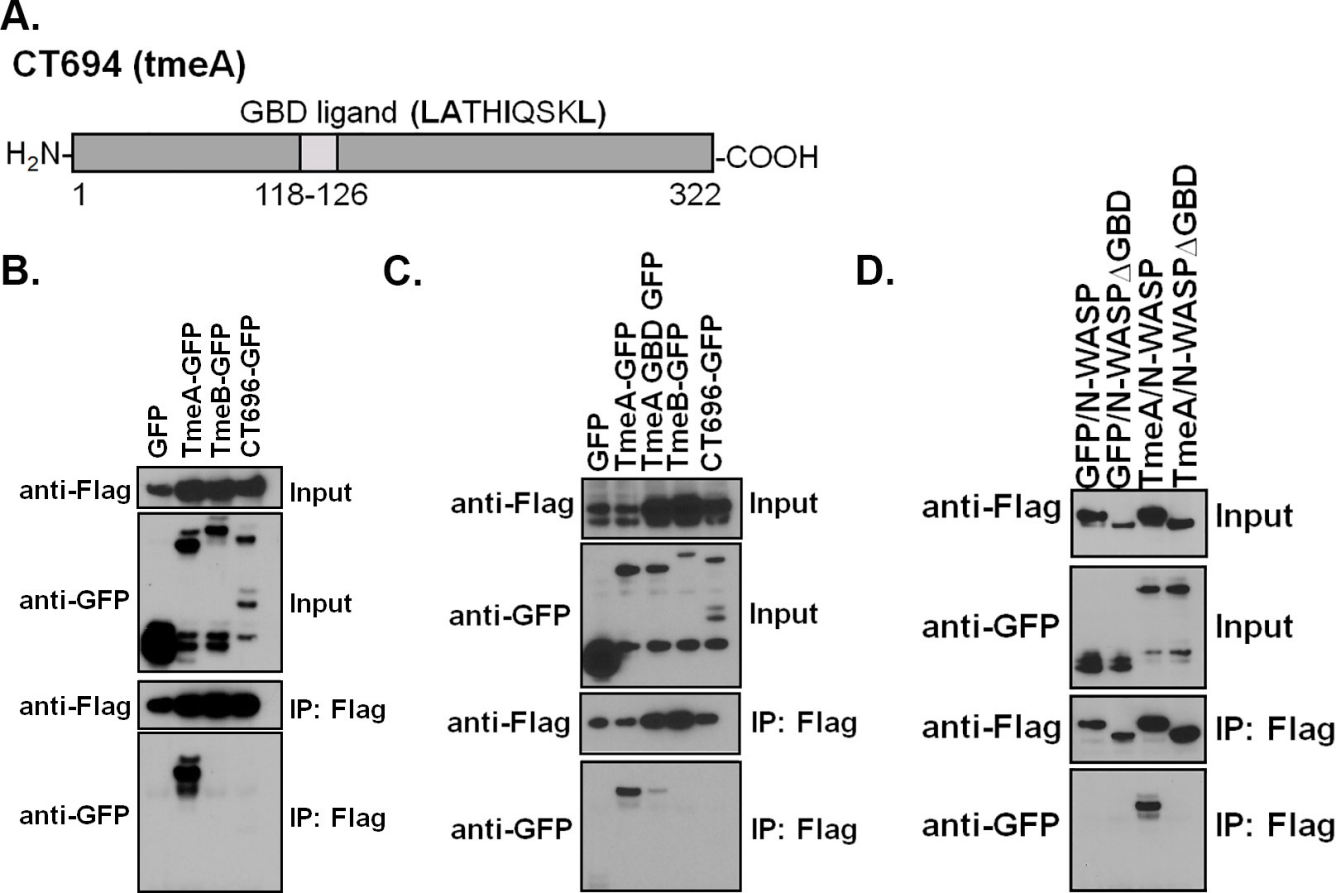
The GTPase binding domain ligand motif of TmeA is necessary for binding to the GTPase binding domain of N-WASP. (**A**) Bioinformatic analysis of TmeA using the Eukaryotic Linear Motif (ELM) identified a Lig_GBD_Chelix1 herein referred to as a GBD ligand motif. (**B**) HeLa cells were transfected with GFP-tagged TmeA (CT694), TmeB (CT695), or CT696 and flag-tagged N-WASP. The flag-tagged fusion was immunoprecipitated and samples were resolved by western blotting. (**C**) Site-directed mutagenesis was used to conservatively mutate (**LA**TH**I**QSK**L➔VS**TH**V**QSK**V**) the GBD ligand motif of TmeA. Immunoprecipitations were conducted as in (**B**). (**D**) The entire GBD of N-WASP was deleted and immunoprecipitations were conducted as in (**B**). (**B-D**) Data are representative of 3 independent experiments.

TmeA is a *C*. *trachomatis* T3SS effector protein that was previously shown to be essential for chlamydial invasion and *in vivo* infection [15]. Yeast-2-hybrid and proximity-based labeling approaches revealed that TmeA binds the large scaffolding protein AHNAK [15,17]. However, TmeA’s role in invasion is independent of its interaction with AHNAK as AHNAK is dispensable for bacterial invasion and AHNAK is recruited to the site of bacterial entry in a manner independent of TmeA [15]. To identify alternative host targets of TmeA, we used a bioinformatics-guided approach to determine whether TmeA might possess eukaryotic-like domains indicative of effector function. Bioinformatic analysis of TmeA using the Eukaryotic Linear Motif (ELM) resource revealed a Lig_GBD_Chelix1 herein referred to as a GBD ligand motif (Fig 1A). This motif is defined by the sequence [ILV][VA][^P][^P][LI][^P][^P][^P][LM]. In TmeA, this helix motif spans amino acid residues 118–126 (**LA**TH**I**QSK**L**) and consists of hydrophobic residues in the 1^st^, 2^nd^, 5^th^, and 9^th^ positions. A similar motif is found in the WCA region of WASP and N-WASP, which, through intramolecular interactions, associates with the GTPase binding domain of itself to prevent activation of the ARP2/3 complex [18]. The Enterohemorrhagic *Escherichia coli* (EHEC) effector protein EspFu possesses 5 and a half nearly identical repeats that contain five identical Lig_GBD_Chelix1 motifs (**VA**QR**L**VQH**L**) that can potently activate N-WASP [20,21]. Thus, our bioinformatic analysis suggest TmeA might bind to N-WASP.

To determine whether TmeA binds to N-WASP, we transfected HeLa cells with flag-tagged N-WASP and GFP alone or GFP-tagged *C*. *trachomatis* effector proteins TmeA (CT694), TmeB (CT695) or CT696. We immunoprecipitated flag-tagged N-WASP and assessed binding of the GFP-tagged effector proteins. Using this approach, we show that TmeA, but not TmeB or CT696, binds to N-WASP (Fig 1B). To confirm that the bioinformatically predicted GBD ligand motif of TmeA is necessary for binding to N-WASP we used site-directed mutagenesis to introduce conservative mutations (**LA**TH**I**QSK**L➔VS**TH**V**QSK**V**) into this motif. As predicted, mutation of the bioinformatically predicted GBD ligand motif of TmeA substantially reduced binding to N-WASP (Fig 1C). These results indicate that TmeA possesses a putative ligand motif that is necessary for binding to N-WASP.

The GBD ligand motif found in N-WASP and the *E*. *coli* T3SS effector protein EspFu binds to the GTPase binding domain of N-WASP. Given that TmeA harbors a similar motif, we hypothesized that TmeA binds to the GBD of N-WASP. To test this hypothesis, the GBD region of N-WASP (residues 190–274) was deleted and TmeA binding was evaluated by coimmunoprecipitation. As shown in Fig 1D, deletion of the GBD region of N-WASP abolished TmeA binding. Collectively our results indicate that the GBD ligand motif of TmeA is required for binding to the GBD of N-WASP.

### TmeA temporally recruits N-WASP and the ARP2/3 complex to the site of *Chlamydia trachomatis* invasion

**Fig 2:**
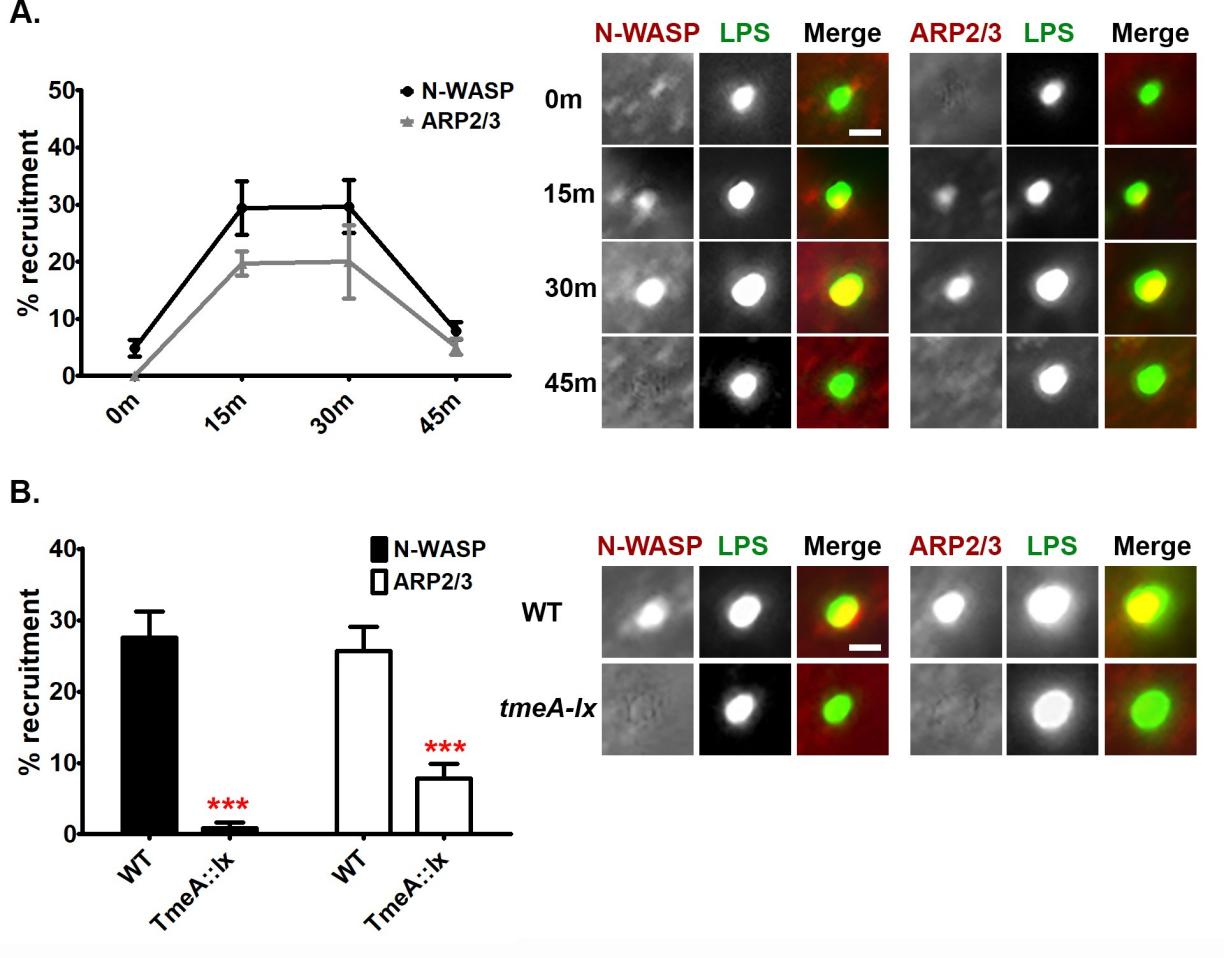
N-WASP and the ARP2/3 complex are temporally recruited to the EB invasion site by TmeA. (**A**) Human cervical cells were infected at a MOI of 5 for 0, 15, 30, or 45min. Chlamydial EBs were stained using an anti-LPS antibody (green) and anti-N-WASP or anti-ARP2/3 (red) antibodies were used to visualize host factors. (**B**) Human cervical cells were infected at a MOI of 5 for 30min with wild-type or *tmeA-lx* and stained as described for (**A**). (**A, B**) Recruitment was assessed from 30 fields of view per experiment. Data are representative of 2 independent experiments. (**B**) Statistical significance was determined using t-test. *** P<0.001. Scale bar is 0.3μm.

To begin to investigate whether the interaction between N-WASP and TmeA could promote *C*. *trachomatis* invasion, we evaluated endogenous N-WASP localization in human cervical cells infected with wild-type (WT) *C*. *trachomatis* serovar L2 for 0, 15, 30, or 45 min. At 15 min post-infection, partial co-localization between *C*. *trachomatis* EBs and N-WASP was noted for 29% of the invading bacteria (Fig 2A). By 30 min post-infection, total co-localization between the EBs and N-WASP was noted for 29% of EBs (Fig 2A, S1 Fig). By 45 min post-infection, the association between EBs and N-WASP dropped to 7.3% (Fig 2A). The association and subsequent dissociation of N-WASP and chlamydial EBs indicates N-WASP likely plays a role during the early stages of chlamydia infection. Recruitment of N-WASP to serovar D was also noted (S2A Fig). To test whether TmeA is necessary for the recruitment of N-WASP to the site of the invading EBs, we evaluated N-WASP localization in the TmeA null strain at 30min post-infection. As shown in Fig 2B, recruitment of N-WASP to the chlamydial EBs was nearly abolished in the absence of TmeA, confirming TmeA is necessary for N-WASP recruitment to the site of the invading bacteria.

N-WASP interacts with the ARP2/3 complex via its WCA domain to promote actin polymerization and branching [18]. To evaluate whether the subcellular localization of endogenous ARP2/3 is also perturbed during chlamydial invasion, human cervical cells were infected with WT *C*. *trachomatis* for 0, 15, 30, and 45 min. Similar to observations with N-WASP, partial or full co-localization between *C*. *trachomatis* EBs and ARP2/3 was noted for ~20% of the invading bacteria at 15 or 30min post-infection, respectively (Fig 2A, S1 Fig). By 45 min post-infection the association between EBs and ARP2/3 had dropped to ~5% (Fig 2A). Thus, the temporal recruitment of the ARP2/3 complex appears to mirror that of N-WASP. Recruitment of ARP2/3 to serovar D was also noted (S2A Fig). To determine whether TmeA impacts the recruitment of the ARP2/3 complex, we evaluated ARP2/3 localization in the TmeA null strain. As shown in Fig 2B, ARP2/3 was still recruited to the TmeA null mutant, albeit recruitment was significantly reduced compared to WT (26% vs. 8%). These results indicate that while TmeA is necessary for N-WASP recruitment, it is only partially required for recruitment of the ARP2/3 complex during *C*. *trachomatis* invasion of nonphagocytic cells.

**Fig 3:**
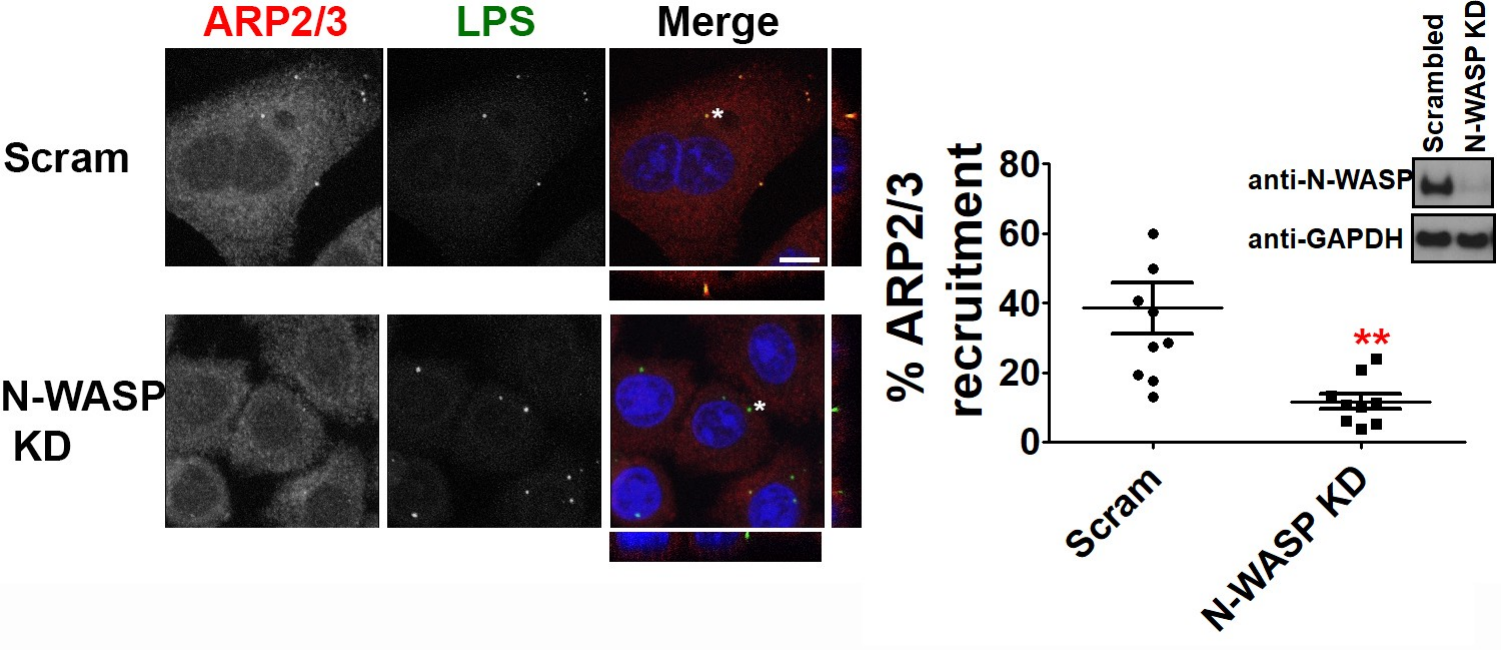
N-WASP is partially required for recruitment of the ARP2/3 complex to the EB invasion site. N-WASP knockdown cells were infected at a MOI of 5 for 30min and stained using anti-ARP2/3 (red) and anti-LPS (green) antibodies. Images were captured by confocal microscopy. The * denotes area used for orthogonal view. Scale bar is 20μm. Recruitment was assessed from 30 fields of view per experiment. Knock down efficiency was confirmed by western blotting. Data are representative of 2 independent experiments. Statistical significance was determined using t-test. ** P<0.01

The fact that the temporal recruitment of the ARP2/3 complex mirrors N-WASP recruitment and that N-WASP is a potent NPF of the ARP2/3 complex prompted us to next ask whether N-WASP is necessary for ARP2/3 recruitment to *C*. *trachomatis* EBs during invasion. Using siRNA, we knocked down the expression of N-WASP and evaluated recruitment of ARP2/3 to chlamydial EBs. As shown in Fig 3, siRNA knockdown of N-WASP reduced ARP2/3 recruitment. Whereas 38% of EBs recruited ARP2/3 in scramble treated cells, only 12% of EBs recruited the ARP2/3 complex in N-WASP knockdown cells (Fig 3). Taken together our results indicate that the chlamydial T3SS effector protein TmeA is required to recruit N-WASP to the bacterial entry site and both TmeA and N-WASP are partially required for the recruitment of the ARP2/3 complex.

### N-WASP and the ARP2/3 complex are essential for *C*. *trachomatis* invasion of nonphagocytic cells

**Fig 4:**
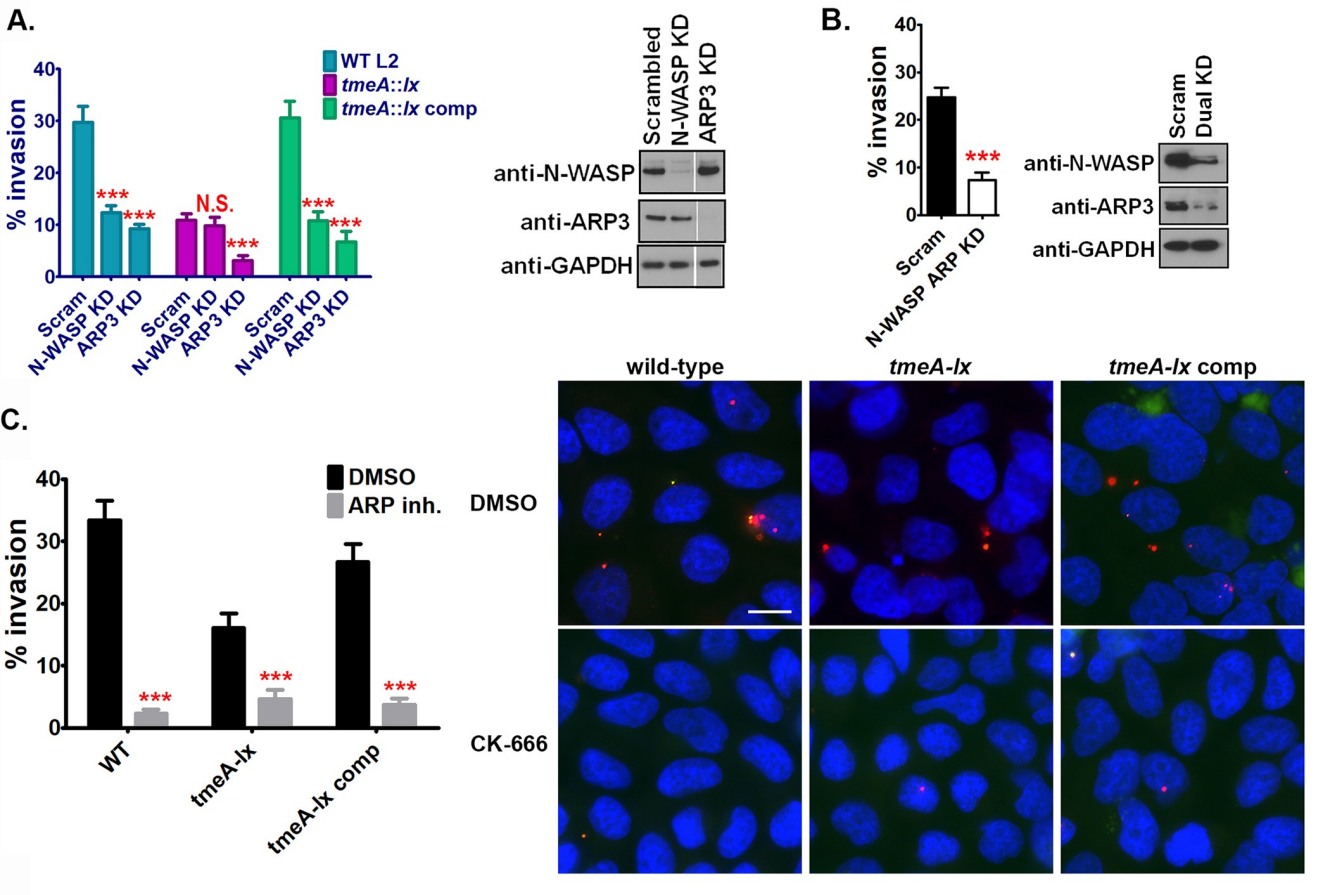
N-WASP and the ARP2/3 complex are required for chlamydial invasion. (**A**) N-WASP and ARP3 knockdown cervical cells were infected at a MOI of 5 for 60min with wild-type, *tmeA-lx*, or *tmeA-lx* comp. The number of internal bacteria was determine using differential immunostaining. Knockdown efficiency was determined by western blotting. (**B**) Dual knockdown of N-WASP and ARP3 was conducted in cervical cells and invasion of wild-type L2 was determined as described in A. (**C**) Human cervical cells were treated with CK-666 and infected at a MOI of 5 for 60min with wild-type, *tmeA-lx*, or *tmeA*::*lx* comp. The number of internal bacteria was determined using differential immunostaining. Scale bar is 20μm. (**A-C**) The number of internal bacteria was enumerated from 30 fields of view per experiment. Data are representative of 2 independent experiments. Statistical significance was determined using One-Way ANOVA (**A**) or t-test (**B, C**). *** P<0.001.

Previous studies have demonstrated that while TmeA is essential for chlamydial invasion, its role in invasion occurs independently of its interaction with AHNAK [15]. Additional studies evaluating the host cell signaling pathways exploited during *C*. *trachomatis* internalization revealed an unprecedented role for N-WASP [26]. Thus, we sought to determine whether it is TmeA’s interaction with N-WASP that promotes chlamydial invasion. siRNA knockdown of N-WASP expression resulted in a 58% reduction in chlamydial invasion whereas siRNA knockdown of ARP3 resulted in a 69% reduction in invasion (Fig 4A). Importantly, a similar reduction in the ability of serovar D to invade human cervical cells was noted (S2B Fig). Strikingly, siRNA knockdown of N-WASP did not further exacerbate the TmeA null mutant phenotype (Fig 4A), suggesting that TmeA is likely the sole chlamydia factor that targets N-WASP. In contrast, siRNA knockdown of ARP3 further reduced the TmeA null mutant’s invasion defect by 70% (Fig 4A). To confirm the synergistic role between N-WASP and the ARP2/3 complex we evaluated the ability of serovar L2 to invade N-WASP/ARP3 dual knockdown cells. As shown in Fig 4B, a 70% reduction in L2 invasion was noted in the dual knockdown cells. A similar invasion defect was noted in the ARP3 knockdown cells (Fig 4A). Collectively, these results suggest that while TmeA uniquely targets N-WASP, chlamydia likely employs other factors that modulate the ARP2/3 complex to ensure pathogen uptake.

To further confirm the importance of the ARP2/3 complex to chlamydial invasion, we used the small molecule inhibitor CK-666, which has been shown to stabilize the inactive form of the ARP2/3 complex, preventing actin nucleation [27]. Human cervical cells were pretreated with the inhibitor for 60min prior to infection, after which cells were infected at a MOI of 5 with each strain. As expected, less internal *tmeA-lx* bacteria were noted compared to wild-type or *tmeA-lx* comp infected cells in control conditions (DMSO). Treatment of cells with the ARP inhibitor (CK-666) reduced the overall number of internal bacteria for each of the 3 strains (Fig 4C) Notably, inhibition of the ARP2/3 complex exacerbated the *tmeA-*lx mutant invasion defect (compare *tmeA-lx* CK-666 to *tmeA-lx* DMSO treated). Importantly, similar numbers of bacteria were observed in all the CK-666 treated cells (compare wild-type, *tmeA-lx*, *tmeA-lx* comp) (Fig 4C). These results are in line with our siRNA studies (Fig 4A) and highlight the critical nature of the ARP2/3 complex for chlamydial invasion. Furthermore, our results suggest that additional chlamydial factors likely target the ARP2/3 complex.

**Fig 5:**
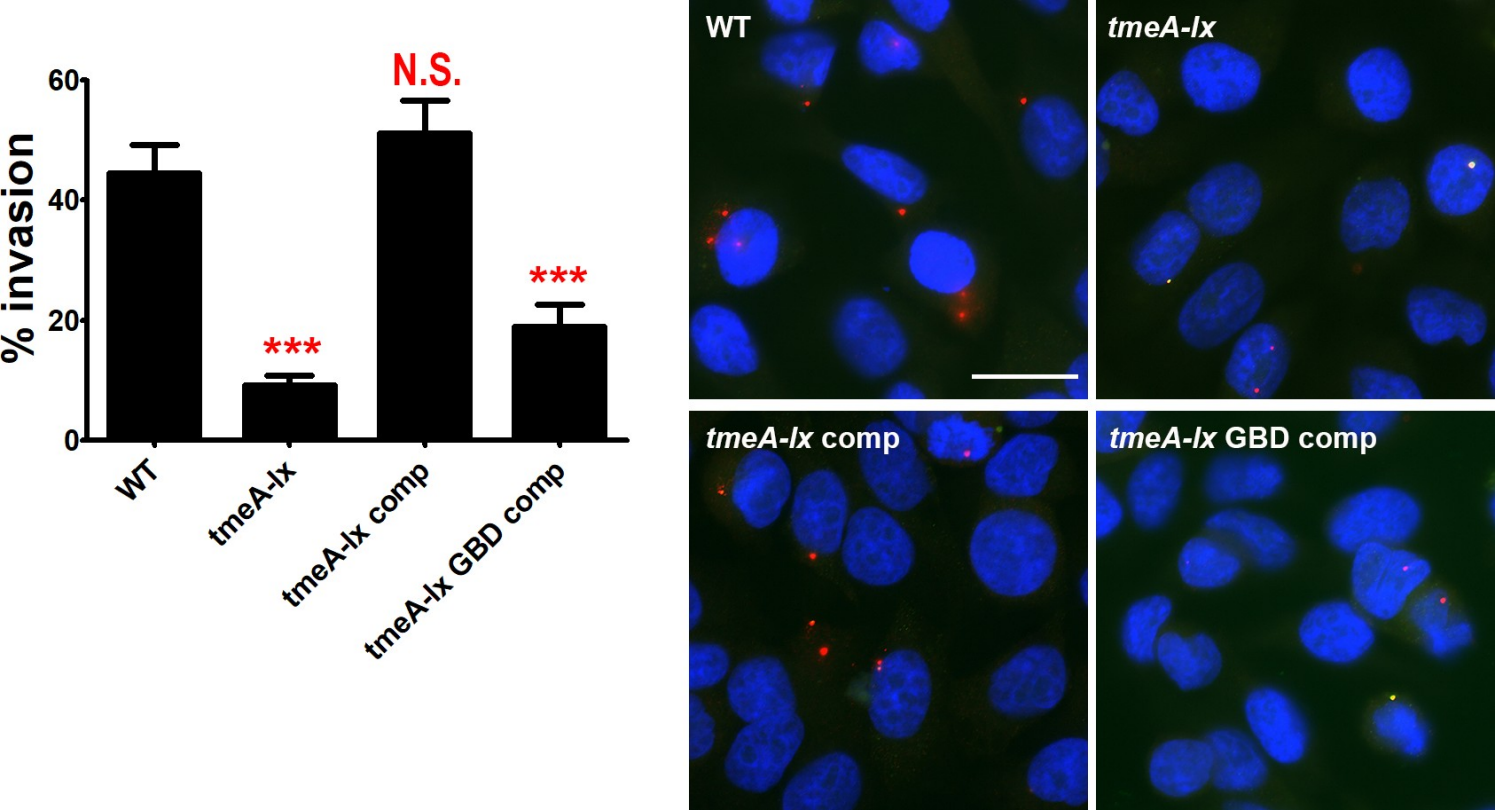
N-WASP/TmeA interactions are required for chlamydial invasion. Human cervical cells were infected at a MOI of 5 for 60min with wild-type, *tmeA-lx*, *tmeA-lx* comp or *tmeA-lx* GBD comp. The number of internal bacteria was determine using differential immunostaining. The number of internal bacteria was enumerated from 30 fields of view per experiment. Data are representative of 2 independent experiments. Statistical significance was determined using One-Way ANOVA. *** P<0.001. Scale bar is 20μm.

Our data indicates that TmeA binds to N-WASP and N-WASP is crucial to chlamydial invasion and recruitment of the ARP2/3 complex. Thus, we hypothesized that it is the inability of TmeA to bind N-WASP that accounts for the TmeA null mutant’s invasion defect. To directly link the TmeA null mutant’s invasion defect to the bacteria’s inability to modulate N-WASP, we complemented the TmeA null mutant with a TmeA variant possessing key mutations in the GBD (**LA**TH**I**QSK**L➔VS**TH**V**QSK**V**) that we showed are necessary to bind N-WASP (Fig 1C). As previously reported [15,16], the TmeA null mutant exhibits a severe invasion defect (Fig 5). As expected, complementation of the TmeA mutant completely restored the bacteria’s ability to invade nonphagocytic cells (Fig 5). Significantly, complementation of the TmeA mutant with a TmeA variant possessing mutations in the GBD ligand motif did not restore the EB invasion defect (Fig 5). Importantly, TmeA and TmeA with a mutant GBD ligand motif were similarly expressed in the null mutant (S3 Fig). Collectively our results indicate that the interaction between TmeA and N-WASP is necessary for chlamydial invasion.

### TmeA is important for filopodia capture of *Chlamydia trachomatis*

**Fig 6:**
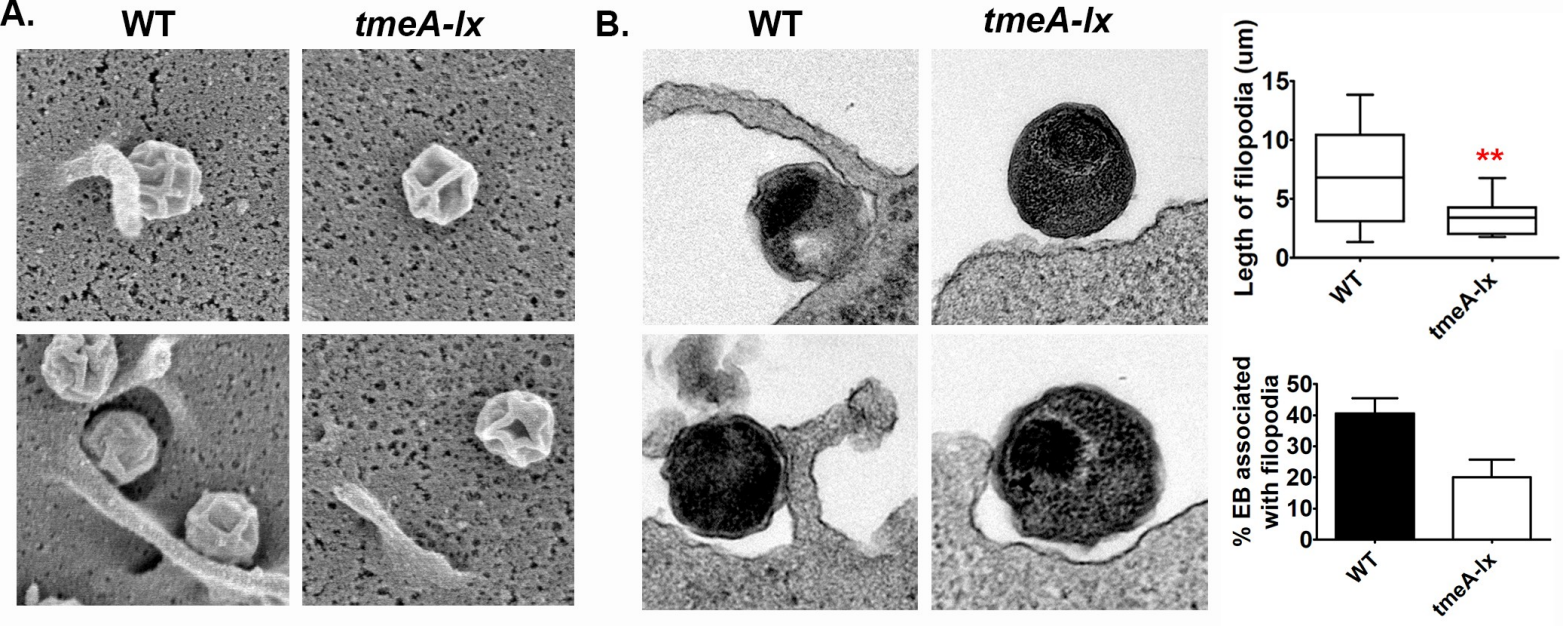
TmeA is partially required for chlamydial association with filopodia. Human cervical cells were infected for 15min at a MOI of 50 with wild-type or *tmeA-lx* and imaged by scanning (A) or transmission (B) electron microscopy. Quantification of EBs associated with filopodia was assessed from two independent experiments with 50 EBs counted per experiment. Length of surface structures were measured to confirm filopodia (top). Bacteria associated with filopodia were compared to total bacteria to determine percent associated with filopodia. Two representative images for each are shown. Scale bar is 200nm.

*C*. *trachomatis* invasion involves the formation of membrane ruffles, pedestal-like structures, and filopodia formation [8,26]. Activation of the NPF N-WASP directs the ARP2/3 complex to polymerize actin, resulting in the formation of actin-rich filopodia structures [18]. Thus, TmeA-mediated manipulation of N-WASP during invasion could account for the formation of filopodia present at the EB entry site. Using scanning (Fig 6A) and transmission (Fig 6B) electron microscopy, we evaluated wild-type and *tmeA-lx* null mutants association with filopodia. Surface structures were measured in Fiji and those between 3–35μm in length (the average size of filopodia) were considered filopodia (Fig 6B top graph). To be considered associated with filopodia, the EBs had to be adjacent to the structure (WT Fig 6B). From duplicate experiment with 50 EBs enumerated per experiment, we observed 41% of wild-type EBs in association with filopodia, however only 20% of *tmeA-lx* EBs were observed in association with filopodia (Fig 6B bottom graph). Notably the tmeA-lx mutant was observed associated with shorter filopodial structures compared to wild-type (Fig 6B top graph). Our results suggest that while TmeA/N-WASP interactions are important for filopodia formation, chlamydia likely employs compensatory mechanisms to ensure filopodia capture of chlamydial EBs during invasion.

### TarP and TmeA target distinct signaling pathways that ultimately converge on the ARP2/3 complex

**Fig 7:**
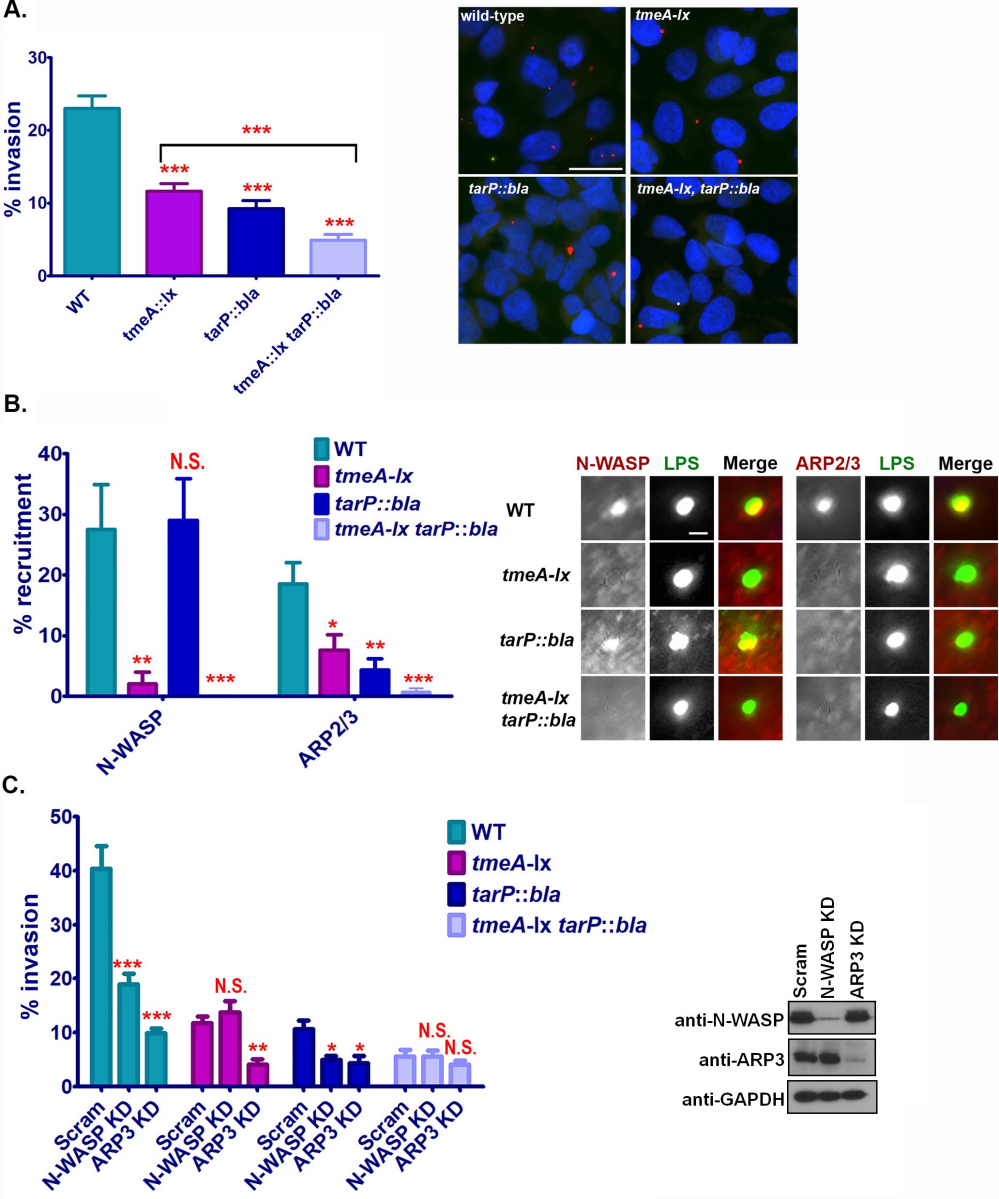
The secreted effector proteins TmeA and TarP target distinct signaling pathways that converge on the ARP2/3 complex. (**A**) Human cervical cells were infected at a MOI of 5 for 60min with wild-type, *tmeA-lx*, *tarP*::*bla*, or *tmeA-lx tarP*::*bla*. The number of internal bacteria were enumerated from 30 fields of view per experiment using differential immunostaining. Scale bar is 20μm. (**B**) Human cervical cells were infected at a MOI of 5 for 30min with wild-type, *tmeA-lx*, *tarP*::*bla*, or *tmeA-lx tarP*::*bla* and chlamydial EBs were stained using an anti-LPS antibody (green) and anti-N-WASP or anti-ARP2/3 (red) antibodies were used to visualize host factors. Recruitment was assessed from 30 fields of view per experiment. Scale bar is 0.3μm. (**C**) N-WASP and ARP3 knock down cervical cells were infected at a MOI of 5 for 60min with wild-type, *tmeA-lx*, *tarP*::*bla*, or *tmeA-lx tarP*::*bla*. The number of internal bacteria was determine using differential immunostaining. (**A-C**) Data are representative of 2 independent experiments. Statistical significance was determined using One-Way ANOVA. *** P<0.001, **P<0.01, *P<0.05.

Collectively our data indicates that TmeA is essential for recruitment of N-WASP to the site of EB invasion. However, TmeA and N-WASP are not the sole determinants of ARP2/3 complex recruitment to chlamydial invasion foci, suggesting that other chlamydial factors may influence ARP2/3 recruitment during invasion. Previous studies indicate that the T3SS effector protein TarP could also manipulate the ARP2/3 complex [8,11,28]. However, until recently the genetic intractability of chlamydia has precluded determining whether TarP influences the ARP2/3 complex during infection. Here we leveraged chlamydial genetics to generate a *tarP*::*bla* single mutant and a *tmeA-lx tarP*::*bla* double mutant to evaluate the contribution of both TarP and TmeA to invasion. As recently reported [13], insertional inactivation of TarP significantly reduced bacterial invasion (Fig 7A). Strikingly, the double mutant had an even more pronounced invasion defect than observed for the *tmeA-lx* or *tarP*::*bla* single mutants (Fig 7A). These results suggest that while TmeA and TarP play essential roles in chlamydia invasion, their roles are not functionally redundant.

To further assess whether the roles of TmeA and TarP are completely distinct during invasion, we evaluated recruitment of N-WASP and ARP2/3 to chlamydial EBs. No difference in N-WASP recruitment was noted between *tarP*::*bla* and wild-type L2 (Fig 7B). Strikingly both the TmeA and TarP mutants were significantly impaired in ARP2/3 recruitment (Fig 7B). Intriguingly ARP recruitment to chlamydial EBs was abolished in the absence of both TmeA and TarP (Fig 7B). Collectively our results indicate that although only TmeA targets N-WASP, both effectors ultimately contribute to the recruitment of the ARP2/3 complex during invasion.

To further confirm that TmeA and TarP target distinct pathways that converge on activation of the ARP2/3 complex, we compared the ability of each mutant to invade human cervical cells treated with siRNA specific for N-WASP or ARP3. While siRNA knockdown of N-WASP did not exacerbate the TmeA mutant entry defect, the TarP mutant was further impaired in its ability to invade cells by 50% (Fig 7C). Importantly, siRNA knockdown of ARP3 further impaired the ability of the TmeA and TarP mutants to invade cells (Fig 7C). Knockdown of N-WASP or ARP3 did not significantly affect the TmeA/TarP mutants ability to invade host cells (Fig 7C).Taken together our results indicate that while both TmeA and TarP are essential for chlamydial invasion, they target distinct cellular pathways that converge on the ARP2/3 complex.

## Discussion

As an obligate intracellular bacterium, invasion of a susceptible host cell is crucial for bacterial replication and initiation of chlamydial disease. To orchestrate this essential event, *C*. *trachomatis* is believed to deliver an array of T3SS effectors into the host cell that induce cytoskeletal rearrangements necessary for pathogen uptake. Proteomic comparison of EBs and RBs has identified several effector proteins that are enriched in EBs and are presumed to be prepackaged at the end of the developmental cycle to initiate new rounds of infection [7]. While insertional inactivation of the early-stage effectors TepP [6,14] and TmeB [15] does not impair invasion, TmeA [15,16] and TarP [13] null mutants are significantly impaired in host cell invasion, highlighting these two effectors as key regulators of chlamydial entry. In this study, we sought to determine how TmeA promotes chlamydial invasion.

To exploit host cell signaling pathways, many bacterial effector proteins possess eukaryotic-like domains that facilitate protein-protein interactions required for host cell subversion [29–33]. Using bioinformatics, we identified a GBD ligand motif in TmeA, a conserved motif found in WASP and N-WASP, which allows for autoinhibition of the NPF [34]. Effector proteins from pathogenic bacteria such as enterohemorrhagic *E*. *coli* (EHEC) EspFu and *Shigella flexneri* IcsA [23,24] similarly possess this motif to exploit N-WASP regulation. Delivery of EspFu into the host cell results in WASP recruitment and pedestal formation to facilitate bacterial attachment to the surface of the host cell and promotes subsequent effector translocation [22,35]. Actin-based motility and cell-to-cell spread of *S*. *flexneri* is mediated by IcsA, which activates N-WASP allowing for direct recruitment of the ARP2/3 complex [23]. Both EspFu and IcsA bind with high affinity to the GBD of N-WASP, releasing the WCA region to bind to the ARP2/3 complex [20,21,23]. Our results indicate that TmeA similarly binds to the GBD of N-WASP. The GBD of N-WASP (residues 190–274) is a large region that possesses a site for Cdc42 binding as well as a site for the WCA of N-WASP to bind to maintain itself in an autoinhibited state. TmeA possesses a single copy of the GBD ligand motif whereas EspFu habors five copies. While one copy it is sufficient to effect N-WASP activation, the additional copies associated with EspFu have been shown to enhance activation [21]. Future studies are needed to determine whether TmeA, possessing one copy of the motif is as efficient of an activator as EspFu. It is intriguing to speculate that the slight sequence difference as well as difference in copy numbers could be a reason for the different attachment consequences for these two pathogens. It is possible that TmeA functions in a manner analogous to that of EspFu and IcsA and binds to the autoinhibitory region to unmask the WCA region for downstream interactions with the ARP2/3 complex. Alternatively, it is possible that TmeA uses a mechanism distinct from that of EspFu and IcsA and instead mimics small GTPase function by binding to the Cdc42 binding region. Whether TmeA mimics the small GTPase Cdc42 or whether it functions as an N-WASP ligand similar to EspFu and IcsA requires further detailed mechanistic study. Regardless, it is intriguing that the small GTPase Rac is essential for chlamydial invasion whereas Cdc42 is dispensable for chlamydial uptake [36], suggesting that *C*. *trachomatis* employs TmeA to circumvent the need for Cdc42 to activate N-WASP function.

**Fig 8:**
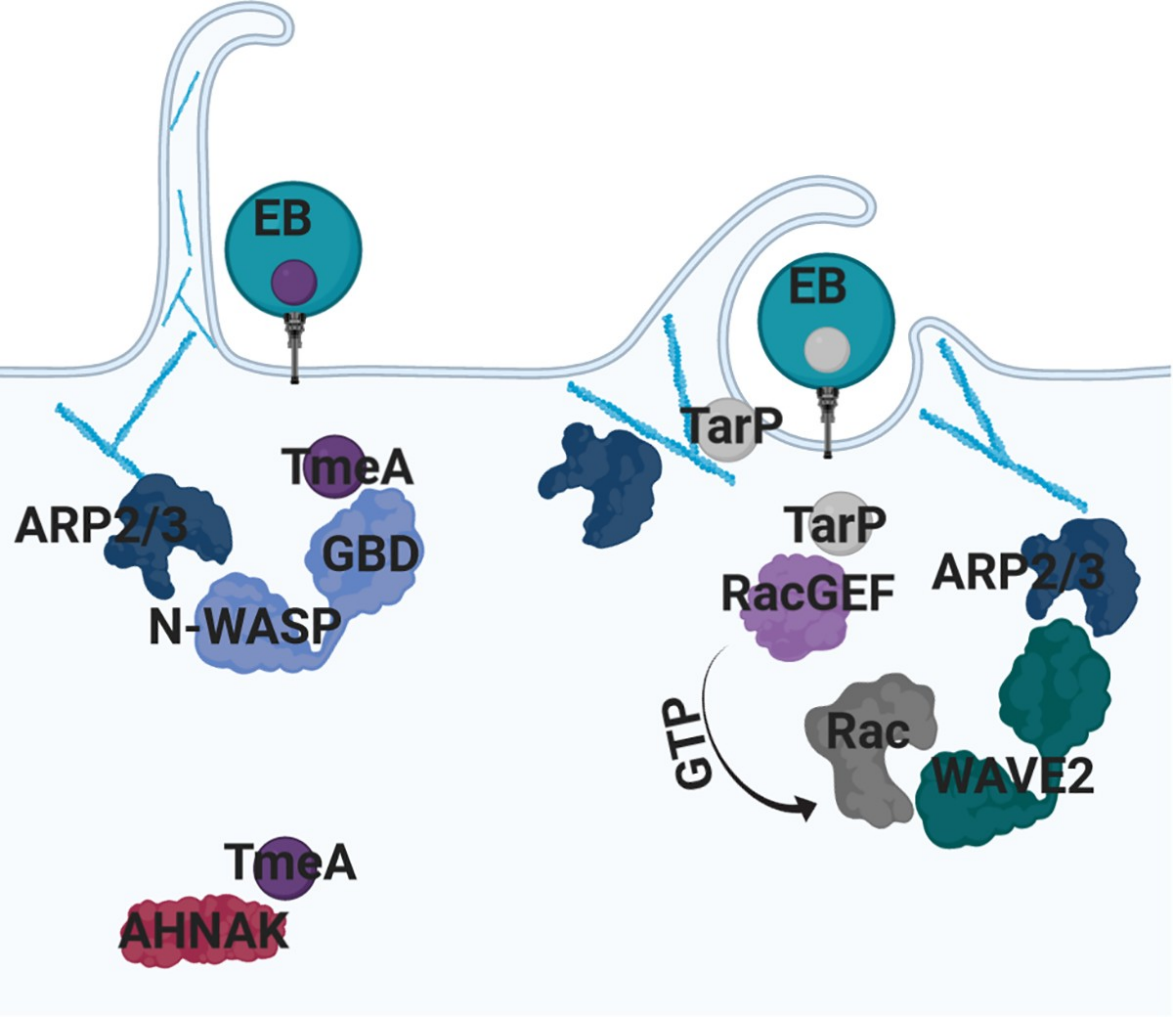
The secreted effector proteins TmeA and TarP target distinct signaling pathways that converge on the ARP2/3 complex. Both TmeA and TarP are type III secretion system effector proteins that are delivered into the host cell prior to chlamydial invasion. TmeA binds to the GTPase binding domain of the nucleation promoting factor N-WASP. Binding of TmeA to N-WASP allows for association with the ARP2/3 complex and induction of actin polymerization and branching events required for filopodia capture of EBs. TmeA also binds to AHNAK, potentially promoting post-invasion functions. TarP is capable of associating with the ARP2/3 complex to increase the rate of actin bundling and polymerization. TarP also binds Rac GEFs, Sos1 and Vav2, to promote ARP2/3 dependent actin polymerization events via the NPF WAVE2.

The use of cryo-electron tomography has recently been used to capture snapshots of how chlamydia EBs are taken up by nonphagocytic cells. These approaches have revealed that *C*. *trachomatis* orients its polarized T3SS to make direct contact with the host cell membrane [37]. Contact between the T3SS and the host cell membrane induces the formation of macropinosomes, phagocytic cups, and actin-rich filopodia [26]. The application of inhibitor-based screens to evaluate the mechanistic underpinnings of chlamydial invasion has highlighted that N-WASP and SNX9 play an unprecedented role in filopodial capture of *C*. *trachomatis* EBs, yet the bacterial factors responsible for driving the changes in the host cell have remained elusive [26]. Building on these findings, our study highlights the secreted effector TmeA as the factor involved in N-WASP recruitment and subsequent filopodia capture of chlamydial EBs. While TmeA was previously shown to interact with AHNAK [15,38], a large scaffolding protein that has been shown to induce F-actin bundling [39], AHNAK is still recruited to the site of EB invasion in the absence of TmeA and no invasion defect was noted in AHNAK-knockout mouse embryonic fibroblasts (MEFs) [15]. These recent studies highlight a major knowledge gap regarding TmeA’s actual role in chlamydial invasion. Results from our study indicate that TmeA promotes chlamydial invasion through manipulation of N-WASP. Several lines of evidence including i) the lack of N-WASP recruitment to the TmeA null mutant, ii) N-WASP knockdown impairs invasion to a degree similar to the TmeA null mutant, and iii) complementation of TmeA with a TmeA variant that is unable to bind N-WASP does not rescue its invasion defect point to TmeA’s role in invasion being through manipulation of N-WASP. However, it is possible that TmeA serves two distinct functions: During invasion, TmeA activates N-WASP to promote ARP2/3 dependent actin polymerization and branching events required for filopodia capture of *C*. *trachomatis* EBs. Following invasion, TmeA could interact with AHNAK to undo the actin-bundling effects induced during the invasion process [40] (Fig 8).

Biochemical studies have shown that TarP promotes actin bundling and polymerization [11] and while it is capable of carrying out these activities in the absence of additional host factors, it is capable of cooperating with the ARP2/3 complex to increase the rate of actin polymerization [28]. TarP also binds to the Rac GEFs Sos1 and Vav2, potentially to drive Rac-dependent activation of the NPF WAVE2 [8,9]. TarP is also presumed to stimulate actin polymerization indirectly through binding and recruitment of FAK [41] and vinculin [42]. While TarP has many diverse functions, genetic studies revealed that the C-terminal F-actin binding and bundling sites are required for TarP-mediated chlamydial invasion [13]. Our observation that chemical inhibition or siRNA knockdown of the ARP2/3 complex exacerbates the invasion defect of the TmeA mutant supports the notion that *C*. *trachomatis* employs other secreted factors that manipulate the ARP2/3 complex. Given the association between TarP and the ARP2/3 complex, we hypothesized that TarP is the other effector that is responsible for ARP2/3 recruitment during invasion. Insertional inactivation of TarP reduced ARP2/3 recruitment to chlamydial invasion sites. Surprisingly, a TmeA/TarP double mutant was still able to invade host cells, albeit at a significantly lower rate. The rate of chlamydial invasion in the absence of these two effector proteins is strikingly similar to that observed when the ARP2/3 complex is inhibited. This suggest that while manipulation of the ARP2/3 complex is the main driver of chlamydial invasion, chlamydia may also exploit ARP2/3 independent pathways for invasion.

While N-WASP and the ARP2/3 complex clearly play an integral role in chlamydial invasion, various screens have identified other factors including cortactin, Abl kinase, WAVE2, SNX9, and COPI [8,26,43–45] that play a role in chlamydial invasion. SNX9 mediates F-actin rearrangements and filopodia formation through associations with N-WASP [46]. Depletion of SNX9 reduces chlamydial invasion and EB association with filopodia [26], analogous to our observations with N-WASP and TmeA. How chlamydia coopts SNX9 remains unclear. While it is possible that a distinct effector manipulates SNX9, it is also possible that TmeA/N-WASP associations might initiate the formation of a scaffold for SNX9 recruitment to the membrane. Equally appealing is the notion that manipulation of other host factors, such as phosphoinositides, may alter SNX9 localization during pathogen invasion similar to what is observed with SopB in *Salmonella* [47]. Regardless of the mechanism employed, it is obvious that chlamydial invasion is a complex event that involves manipulation of multiple host factors to coordinate the necessary cytoskeletal modifications during pathogen invasion.

In conclusion, we provide evidence that *C*. *trachomatis* employs TmeA to directly manipulate N-WASP, promoting recruitment of the ARP2/3 complex to the site of invasion, which we hypothesize coordinates membrane remodeling events necessary for pathogen infiltration. Furthermore, although TmeA-mediated chlamydial invasion triggers pathways independent of TarP, the activity of both of these effectors ultimately influence ARP2/3 recruitment to the site of invasion (Fig 8). We predict that a holistic understanding of how *C*. *trachomatis* uses secreted effector proteins to manipulate host membrane dynamics will highlight host pathways crucial for invasion that can be subsequently targeted to block uptake of this and potentially other significant human pathogens.

## Methods

### Bacterial and cell culture

*Chlamydia trachomatis* serovar L2 (LGV 434/Bu) and serovar D/UW-3/CX) was propagated in HeLa 229 cells (American Type Culture Collection) and EBs were purified using a Gastrografin density gradient [48]. HeLa cells (ATCC) were propagated in RPMI 1640 media with L-Glutamine (ThermoFisher Scientific) supplemented with 10% Fetal Bovine Serum (FBS) at 37°C and 5% CO_2_. Human cervical keratinocytes (HCK) [49] were propagated in keratinocyte serum free media (K-SFM) supplemented with bovine pituitary extract (BPE) and epidermal growth factor (EGF) (ThermoFisher Scientific) at 37°C and 5% CO_2_.

### Complementation of *tmeA-lx*

*tmeA* was PCR amplified from *C*. *trachomatis* L2/434/Bu DNA and fused to flag-tag using gene specific primers. The PCR product was digested and ligated into the NotI/SalI site of pBomb4-tet-mcherry [50,51]. The integrity of the construct was confirmed by DNA sequencing (McLab). pBomb4-tet-TmeA flag was used as a template to generate pBomb4-tet-TmeA GBD (**LA**TH**I**QSK**L➔VS**TH**V**QSK**V**) flag by GenScript. Both constructs were transformed into *tmeA-lx* as previously described [52]. Expression of the flag-tagged fusion protein was confirmed by western blotting.

### Generation of *C*. *trachomatis tarP*::*bla*

The intron was retargeted for *C*. *trachomatis* 434/Bu CT456 (*tarP*) using the TargeTron computer algorithm (TargeTronics). Insertion sites with the highest score and the closest proximity to the 5′ ATG start codon were selected. The intron was retargeted and amplified using a Qiagen core PCR kit (Qiagen). The PCR product was cloned into the BsrGI/HindIII site of pACT, and the ligated plasmid was transformed into methylation-deficient *Escherichia coli* K-12 ER2925 (New England BioLabs). The integrity of all constructs was verified by sequencing. *C*. *trachomatis* serovar L2 was transformed with each TargeTron construct as previously described [50,52,53]. CT456 was amplified with gene specific primers and sequenced to verify intron orientation.

### Coimmunoprecipitation

HeLa cells were co-transfected with flag-tagged N-WASP or N-WASPΔGBD and GFP-tagged TmeA, TmeA GBD, TmeB, CT696, or GFP alone. Host cells were lysed with eukaryotic lysis solution (50mM Tris HCl, pH7.4, 150mM NaCl, 1mM EDTA, and 1% Triton-X 100) and flag-tagged N-WASP was immunoprecipitated using M2 affinity resin (Millipore Sigma). Samples were analyzed by SDS-PAGE and transferred to PVDF membranes. Membranes were probed with anti-flag or anti-GFP antibodies. Results were collected from at least 3 independent experiments.

### siRNA knockdown

HCK cells were seeded on glass coverslips in 24-well plates at 5X10^4^. After 24h the cells were transfected with SmartPool siRNA for N-WASP or ARP3 per the manufactures guidelines (Dharmacon). At 48h post-transfection, cells were infected on ice at a MOI of 5 with each strain. After 30min the inoculum was removed, the cells were washed twice with 1X PBS, and subsequently incubated at 37°C with 5% CO_2_ for 60min. Cells were fixed with 4% formaldehyde and were differentially immunostained as previously described [49]. Images were collected using a Nikon Ti2 immunofluorescent microscope. The number of internal bacteria (single stained) and number of host cells (DAPI stained) were enumerated from at least 30 images per experiment with at least 2 independent experiments. siRNA knockdown was confirmed using western blotting.

### Immunofluorescence and confocal microscopy

HCK cells were seeded on glass coverslips in 24-well plates to 1X10^5^. After 24h the cells were placed on ice for 10min. Cells were infected on ice at a MOI of 5 with each strain. After 30min the inoculum was removed, the cells were wash twice with 1X PBS, and subsequently incubated at 37°C with 5% CO_2_. At the designated time, cells were fixed with 4% formaldehyde and permeabilized with 0.1% Triton-X 100. Cells were stained with anti-chlamydia LPS (Novus) and anti-N-WASP (Novus) or anti-ARP2/3 (Novus). Dylight-488 or Dylight-594 secondaries (ThermoFisher) and DAPI (ThermoFisher) was used to stain the nucleus. Images were collected using a Nikon Ti2 immunofluorescent microscope or Leica SPE confocal. At least 30 images were collected per experiment with at least 2 independent experiments. Colocalization between LPS and N-WASP or ARP2/3 was measured using the CoLoc2 function in Fiji.

### Invasion assay

To induce expression of TmeA-flag or TmeA GBD flag, cultures were induced with aTc at 40hr post-infection. At 48hr post-infection, host cells were lysed and the bacteria were purified by gastrograffin density gradient [48]. HCK cells were seeded on glass coverslips in 24-well plates at 1X10^5^. After 24h the cells were placed on ice for 10min. Cells were infected on ice at a MOI of 5 with each strain. After 30min the inoculum was removed, the cells were wash twice with 1X PBS, and subsequently incubated at 37°C with 5% CO_2_ for 60min. Cells were fixed with 4% formaldehyde and differential immunostaining was conducted as previously described [49]. Images were collected using a Nikon Ti2 immunofluorescent microscope. The number of internal bacteria (single stained) and number of host cells (DAPI stained) was enumerated from at least 30 images per experiment with at least 2 independent experiments.

### Transmission and scanning electron microscopy

HCK cells, seeded at 2X10^6^/well, were infected at a MOI of 50 with wild-type L2 or *tmeA-lx* for 15min. Cells were fixed in 2.5% glutaraldehyde overnight at 4°C. Cells were then post fixed in 1% osmium tetroxide with 0.8% potassium ferricyanide. Specimens destined for transmission electron microscopy were en bloc stained in uranyl acetate, dehydrated in a graded ethanol series and embedded in Eponate12 resin (Ted Pella, Inc.) Thin sections (70 nm) were cut using a Leica UC6 ultramicrotome (Leica Microsystems) and were viewed on a JEOL JEM 1230 transmission electron microscope. Digital images were acquired with a Gatan UltraScan 1000 2k x 2k CCD camera. Surface structures were measured in Fiji and those between 3–35μm in length (the average size of filopodia) were considered filopodia. To be considered associated with filopodia, the EBs had to be adjacent to the structure. Fifty EBs were enumerated per experiment and TEM was conducted in duplicate. Following glutaraldehyde and osmium fixation, samples destined for scanning electron microscopy imaging were dehydrated in a graded ethanol series, transitioned to hexamethydisilazane and air-dried overnight. The samples were then mounted, sputter coated with 60/40 gold/palladium and imaged in an Hitachi S-4800 scanning electron microscope.

### Data analysis

When required, statistical analysis was conducted using GraphPad Prism software. One-Way ANOVA or T-test was used and generated a statistical difference of p<0.001 (***), p<0.01 (**), or p<0.05 (*). When applicable, Tukey or Dunnett was used as a post-test.

## Supporting information

S1 Fig N-WASP and ARP2/3 are recruited to *C. trachomatis* L2 EBs. Human cervical cells were infected at a MOI of 5 for 30min. Chlamydial EBs were stained using an anti-LPS antibody (green) and anti-N-WASP or anti-ARP2/3 (red) antibodies were used to visualize host factors. Images were acquired by confocal microscopy. * denotes area used for orthogonal view. Scale bar is 20μm.
(TIF)

S2 Fig N-WASP and ARP2/3 are important for serovar D infection. **(A)** Human cervical cells were infected at a MOI of 5 for 30min. *C*. *trachomatis* serovar D EBs were stained using an anti-LPS antibody (green) and anti-N-WASP or anti-ARP2/3 (red) antibodies were used to visualize host factors. Images were acquired by confocal microscopy. * denotes area used for orthogonal view. (B) N-WASP and ARP3 knockdown cervical cells were infected at a MOI of 5 for 60min with serovar D. The number of internal bacteria was determine using differential immunostaining. Knockdown efficiency was determined by western blotting. Data are representative of 2 independent experiments. Statistical significance was determined using One-Way ANOVA. *** P<0.001.
(TIF)

S3 Fig Expression of TmeA and TmeA GBD in *Chlamydia trachomatis*. The *tmeA-lx* comp and *tmeA-lx* GBD comp cells were used to infect HeLa cells for 40h, after which expression of the TmeA flag fusion protein was induced with aTc for 8h. HeLa cells were lysed and EBs were isolated and analyzed for expression of the fusion protein by Western blotting. Anti-HSP was used to ensure equal loading.
(TIF)
