# Supplementary figures and images for: The Chlamydia trachomatis secreted effector TmeA hijacks the N-WASP-ARP2/3 actin remodeling axis to facilitate cellular invasion
Source: PLoS Pathog. 2020 Sep 18;16(9):e1008878. doi: 10.1371/journal.ppat.1008878 (PMC7526919; doi:10.1371/journal.ppat.1008878)

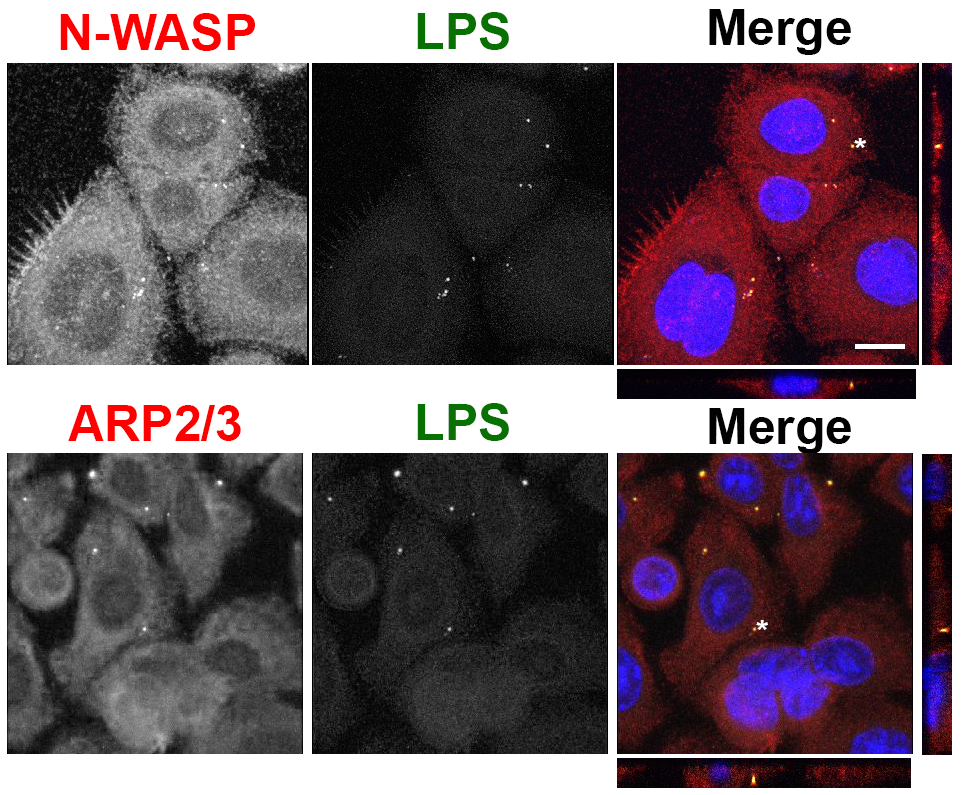

Supplement: S1 Fig — Human cervical cells were infected at a MOI of 5 for 30min. Chlamydial EBs were stained using an anti-LPS antibody (green) and anti-N-WASP or anti-ARP2/3 (red) antibodies were used to visualize host factors. Images were acquired by confocal microscopy. * denotes area used for orthogonal view. Scale bar is 20μm. (TIF) [file ppat.1008878.s001.tif]

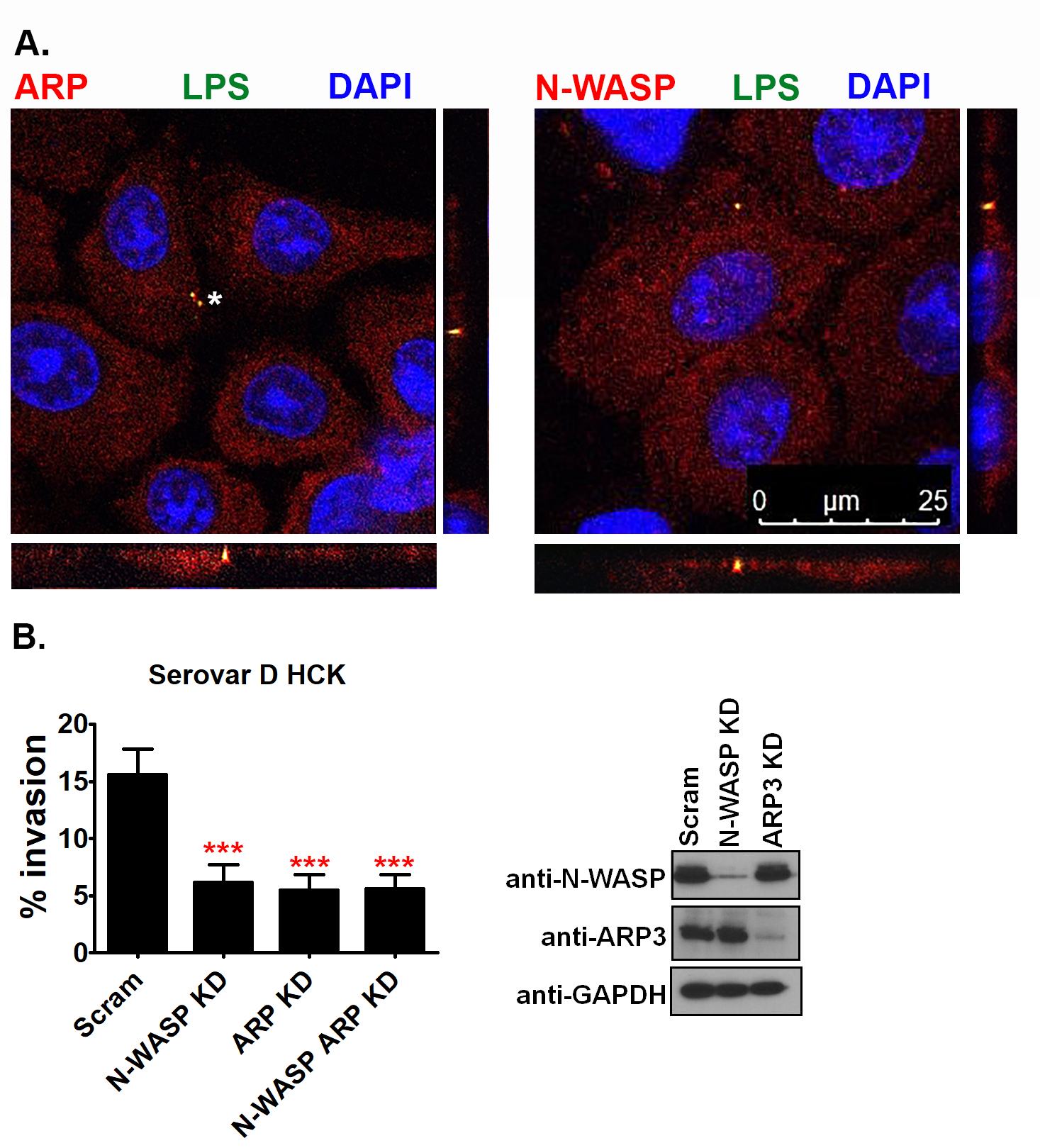

Supplement: S2 Fig — (A) Human cervical cells were infected at a MOI of 5 for 30min. C. trachomatis serovar D EBs were stained using an anti-LPS antibody (green) and anti-N-WASP or anti-ARP2/3 (red) antibodies were used to visualize host factors. Images were acquired by confocal microscopy. * denotes area used for orthogonal view. (B) N-WASP and ARP3 knockdown cervical cells were infected at a MOI of 5 for 60min with serovar D. The number of internal bacteria was determine using differential immunostaining. Knockdown efficiency was determined by western blotting. Data are representative of 2 independent experiments. Statistical significance was determined using One-Way ANOVA. *** P<0.001. (TIF) [file ppat.1008878.s002.tif]

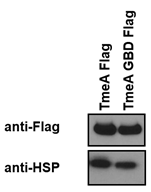

Supplement: S3 Fig — The tmeA-lx comp and tmeA-lx GBD comp cells were used to infect HeLa cells for 40h, after which expression of the TmeA flag fusion protein was induced with aTc for 8h. HeLa cells were lysed and EBs were isolated and analyzed for expression of the fusion protein by Western blotting. Anti-HSP was used to ensure equal loading. (TIF) [file ppat.1008878.s003.tif]
